# Exploring *PDLIM2* as a prognostic biomarker and therapeutic target in lung adenocarcinoma

**DOI:** 10.1038/s41416-026-03570-3

**Published:** 2026-08-08

**Authors:** Karam Ashouri, Fan Sun, Harris Krause, Andrew Elliott, Stephen V. Liu, Patrick C. Ma, Balazs Halmos, Gutian Xiao, Ari Vanderwalde, Jorge J. Nieva, Zhaoxia Qu

**Affiliations:** 1 Norris Comprehensive Cancer Center, Keck School of Medicine, University of Southern California, Los Angeles, CA USA; 2 UPMC Hillman Cancer Center, University of Pittsburgh School of Medicine, Pittsburgh, PA USA; 3 Caris Life Sciences, Phoenix, AZ USA; 4 Georgetown University, Washington, DC USA; 5 Penn State Cancer Institute, Penn State College of Medicine, Hershey, PA USA; 6 Montefiore Medical Center, Bronx, New York, NY USA; 7 Department of Immunology and Immune Therapeutics, Keck School of Medicine, University of Southern California, Los Angeles, CA USA; 8 Hastings Center for Pulmonary Research, Keck School of Medicine, University of Southern California, Los Angeles, CA USA

**Keywords:** Biomarkers, Non-small-cell lung cancer, Cancer immunotherapy

## Abstract

**Background:**

While PDZ-LIM domain-containing protein (PDLIM2) suppresses lung cancer, its clinical significance and therapeutic potential remain to be fully explored.

**Methods:**

Next-generation sequencing and immunohistochemistry of programmed death ligand 1 (PD-L1) were performed on lung adenocarcinoma (LUAD) tissues from 15,765 patients. *PDLIM2* gene therapy was tested in syngeneic LUAD mouse models using intravenous nano-complexed plasmid DNA, alone or with chemoimmunotherapy.

**Results:**

*PDLIM2*-high tumors were more common in primary/local biopsies versus metastatic samples (73.1% vs 53.0%; *p* < 0.001). High *PDLIM2* expression was linked to lower mutation rates in *RB1*, *TP53*, *SMARCA4, STK11*, and *KEAP1*, but increased *EGFR* mutations (all *p* < 0.01). *PDLIM2*-high tumors also showed increased immune cell infiltration, T cell-inflamed scores, and PD-L1 positivity (all *p* < 0.008). High *PDLIM2* was associated with improved survival (24.1 vs 18.1 months; *p* < 0.001, HR = 0.84) and remained prognostic in multivariate analysis (HR = 0.88, 95% CI 0.83–0.93), as well as with longer pembrolizumab time, especially with platinum-based therapy (*p* = 0.012, HR = 0.867). In two LUAD mouse models, nanoPDLIM2 improved median overall survival versus chemoimmunotherapy alone (10–12.5 vs 8–9 days; N = 8; *p* = 0.0001, 0.0003).

**Conclusion:**

*PDLIM2* is a promising prognostic biomarker in LUAD linked to immune-receptive tumors. Preclinically, *PDLIM2*-based therapy enhances chemoimmunotherapy efficacy, though its predictive role warrants further evaluation.

## Introduction

Lung cancer remains the leading cause of cancer-related deaths among both men and women in the U.S. with a 5-year survival rate of only 27% [1]. Blockade of the interaction between the immune checkpoint PD-1 (also known as CD279) and PD-L1 (also known as B7-H1 or CD274) using PD-1 or PD-L1 antibodies is now part of the standard treatment for advanced *EGFR*/*ALK* wild type non-small cell lung cancer [2–4]. Given the synergy demonstrated in preclinical and clinical trials, chemoimmunotherapy is an established treatment option that combines immunotherapy with traditional chemotherapy [5–9]. Tumor PD-L1 expression is the most commonly used biomarker of ICI response; however, its predictive value in lung cancer is poor [10]. Therefore, it is essential to identify additional biomarkers for ICI response, understand the mechanisms underlying ICI resistance in lung adenocarcinoma (LUAD), and explore new combination therapies to improve treatment outcomes.

*PDLIM2* is a tumor suppressor implicated in the pathogenesis of various cancers [5, 6, 11–18]. In non-small cell lung cancer (NSCLC) including LUAD, *PDLIM2* expression is frequently reduced, and this reduction is associated with poor clinical outcomes [5, 6]. Functionally, PDLIM2 has been shown to suppress tumor cell growth, migration, and invasion in both in vitro and in vivo models [5, 6]. Furthermore, *PDLIM2* restoration by nano-delivery of exogenous *PDLIM2* (nanoPDLIM2) or pharmacological induction of endogenous *PDLIM2* enhances the sensitivity of chemotherapy and immunotherapy in a mouse model of primary LUAD [5, 6].

The tumor-suppressive effects of *PDLIM2* are mediated through multiple mechanisms [18]. One key mechanism involves the negative regulation of the NF-κB and STAT3 signaling pathways [5, 6, 11–13, 19, 20]. NF-κB and STAT3 are two master transcription factors that play a central role in inflammation, metabolic reprogramming, cell survival, and tumorigenesis [21–30]. A recent study suggests that instead of being claimed as a ubiquitin ligase (E3), PDLIM2 acts as the novel ubiquitin ligase enhancer (E5) to stabilize and chaperone the E3 SCF^β-TrCP^ to ubiquitinate nuclear RelA for proteasomal degradation, therefore suppressing NF-κB activity [31]. RelA, also known as p65, is the prototypical member of NF-κB that is consistently activated in lung and many other cancers [5, 21, 22].

Although the significance of *PDLIM2* in LUAD has been studied—mainly in preclinical models, where its repression is linked to therapeutic resistance and immune evasion—its clinical relevance, especially in relation to specific molecular alterations and the tumor immune microenvironment, remains incompletely understood. Particularly, its role in patients’ response to immunotherapy has not been established. Moreover, the efficacy of *PDLIM2*-based therapies including the inventive nanoPDLIM2 is yet to be tested in preclinical models of aggressive lung cancer and metastatic LUAD in particular. This manuscript aims to bridge these critical gaps by leveraging a clinical dataset of LUAD patients to evaluate the association of *PDLIM2* expression with tumor molecular alterations, immune cell infiltration, and clinical outcomes. Two syngeneic mouse models of metastatic LUAD are also employed to examine if nanoPDLIM2 can improve the efficacy of chemoimmunotherapy for the better treatment of LUAD metastasis. These studies will provide critical insights into the potential of *PDLIM2* as a prognostic biomarker and therapeutic target for LUAD.

## Material and methods

### Human LUAD data

Next generation sequencing (NGS) of DNA (592-gene panel or whole exome) and RNA (whole transcriptome) was performed for LUAD (N = 15765) patient tumors submitted to Caris Life Sciences (Phoenix, AZ, USA). Patients with histologically confirmed non-small cell lung adenocarcinoma were included in the study, with other subtypes excluded. Mutations were defined as pathogenic single nucleotide variants (SNVs) and indels (insertions or deletions). Samples were stratified by *PDLIM2* expression quartiles (in transcripts per million [TPM]) for all tumors (Q1 to Q4, the lowest to highest). High tumor mutational burden (TMB-high) was set as ≥10 mutations per Mb. Cell infiltration in the tumor microenvironment was estimated by QuantiSEQ. Gene expression profiles were analyzed for transcriptional signatures predictive of response to immunotherapy (T cell-inflamed). Real-world overall survival was assessed from insurance claims data and Kaplan-Meier estimates were calculated for molecularly defined subpopulations. Tumors were also stained immunohistochemically (IHC) with PD-L1 antibodies (clone 22C3, RRID:AB_2687655, Agilent Technologies Inc., Santa Clara, CA, USA) and those with tumor proportion score (TPS) ≥1% were defined as Positive (+). Mann-Whitney U and X^2^/Fisher-Exact tests were applied where appropriate, with *p*-values adjusted for multiple comparisons (*p* < 0.05).

### DNA NGS

NGS was performed on genomic DNA isolated from formalin-fixed paraffin-embedded (FFPE) tumor samples using the NextSeq or NovaSeq 6000 platforms (Illumina, Inc., San Diego, CA). For NextSeq sequenced tumors, a custom-designed SureSelect XT assay was used to enrich 592 whole-gene targets (Agilent Technologies, Santa Clara, CA). For NovaSeq sequenced tumors, more than 700 clinically relevant genes at high coverage and high read-depth were used, along with another panel designed to enrich for an additional >20,000 genes at lower depth. All variants were detected with > 99% confidence based on allele frequency and amplicon coverage, with an average sequencing depth of coverage of > 500 and an analytic sensitivity of 5%. Prior to molecular testing, tumor enrichment was achieved by harvesting targeted tissue using manual microdissection techniques. Genetic variants identified were interpreted by board-certified molecular geneticists and categorized as ‘pathogenic,’ ‘likely pathogenic,’ ‘variant of unknown significance,’ ‘likely benign,’ or ‘benign,’ according to the American College of Medical Genetics and Genomics (ACMG) standards. When assessing mutation frequencies of individual genes, ’pathogenic,’ and ‘likely pathogenic’ were counted as mutations.

### RNA NGS

Qiagen RNA FFPE tissue extraction kit was used for RNA extraction, and the RNA quality and quantity were determined using the Agilent TapeStation. Biotinylated RNA baits were hybridized to the synthesized and purified cDNA targets, and the bait-target complexes were amplified in a post capture PCR reaction. The Illumina NovaSeq 6500 was used to sequence the whole transcriptome from patients to an average of 60 M reads. Raw data was demultiplexed by Illumina Dragen BioIT accelerator, trimmed, counted, PCR-duplicates removed and aligned to human reference genome hg19 by STAR aligner. For transcription counting, transcripts per million values were generated using the Salmon expression pipeline.

### MSI (microsatellite instability) and MMR (mismatch repair) test

A combination of test platforms was used to determine the MSI or MMR status of the tumors profiled, including IHC (MLH1, M1 antibody; MSH2, G2191129 antibody; MSH6, 44 anti-body; and PMS2, EPR3947 antibody (Ventana Medical Systems, Inc., Tucson, AZ) and NGS ( > 2800 target microsatellite loci were examined and compared to the reference genome hg19 from the University of California, Santa Cruz (UCSC) Genome Browser database). The platforms generated highly concordant results as previously reported (Vanderwalde 2018 Cancer Medicine) and in the rare cases of discordant results, the status was determined by IHC.

### Real-world survival analysis

Real-world overall survival (OS) was inferred from insurance claims data and calculated from date of biopsy collection or first of treatment time to last contact, while time-on-treatment (TOT) was calculated from first to last of treatment time. Hazard ratio (HR) was calculated using the Cox proportional hazards model, and *p* values were calculated using the log-rank test. Given the large number of samples, no power analysis was conducted.

### Compliance statement

This retrospective study was conducted under Caris Life Sciences’ Research Data Banking protocol, which was reviewed and granted IRB exemption by the WCG IRB. The study adhered to the ethical guidelines of the Declaration of Helsinki, the Belmont Report, and the U.S. Common Rule.

### Animals and cell lines

BALB/c mice (RRID:IMSR_JAX:000651) and FVB/N mice (RRID:IMSR_JAX:001800) were originally purchased from The Jackson Laboratory and maintained under specific pathogen-free conditions and used according to protocols approved by Institutional Animal Care and Use Committee (IACUC) of the University of Pittsburgh. Mouse lung cancer cell lines Madison 109 (MAD109) and LAP0297 were kindly provided by Alan L Epstein (University of Southern California, Los Angeles, CA, USA) and Dai Fukumura/Peigen Huang (Massachusetts General Hospital and Harvard Medical School, Boston, MA, USA), respectively [32, 33]. The cell lines were cultured in Dulbecco’s modified Eagle’s medium (DMEM) supplemented with 10% fetal bovine serum (FBS) and penicillin/streptomycin (Invitrogen, Carlsbad, CA, USA) and were cultured at 37 °C in a humidified 5% CO_2_ incubator. The cell lines were authenticated by short tandem repeat profiling and tested negative for Mycoplasma.

### nanoPDLIM2 preparation

The in vivo-jetPEI nanoparticles (Polyplus Transfection, New York, NY, USA) and plasmid DNA complexes at a nitrogen-to-phosphate ratio of 8 (N/P = 8) were prepared according to the manufacturer’s instructions. Briefly, 25 μg of pCMV4-myc-PDLIM2 or empty pCMV4 vector in 100 μl of 5% glucose solution was mixed with 4 μl in vivo-jetPEI reagent that was freshly diluted in 5% glucose solution in a final volume of 100 μl. After 15 minutes of incubation at room temperature, the mixed solution was injected intravenously (i.v.) via the tail vein at a dose of 200 μl per mouse [34].

### Syngeneic mouse models of metastatic LUAD

Six-week-old female BALB/c or FVB/N mice were challenged subcutaneously (s.c.) in the right flank with 10^6^ MAD109 or LAP0297 lung tumor cells, respectively. Sixteen days post-tumor cell inoculation, subcutaneous tumors were removed. Animals were randomly allocated to treatment groups using a computer-generated randomization sequence to minimize selection bias. Inclusion criteria for mice were healthy 6-week-old females with a body weight 18-22 g. Exclusion criteria included signs of illness, abnormal behavior, or weight loss >10% during acclimatization period. One cohort of mice were i.v. injected with nanoPDLIM2 or nano-vector as described above ten days post subcutaneous tumor removal. A second cohort of mice were intraperitoneally (i.p.) injected with the chemotherapeutic drugs carboplatin and paclitaxel (AdipoGen Life Sciences, San Diego, CA, USA) at doses of 30/15 mg per kg body weight eleven days post subcutaneous tumor removal, followed by i.p. injection of PD-1 antibody (BioXCell, West Lebanon, NH, USA) at a dose of 200 µg per mouse starting on the second day after chemotherapeutic drugs treatment for three consecutive days. Some mice received both treatments. All mice were monitored daily. No attrition occurred as all animals completed the study as planned. Kaplan-Meier estimates were used to calculate mouse survival and Log-rank (Mantel-Cox) test was used to determine the potential survival difference among different mouse groups [35, 36].

### Study approval

We have complied with all relevant ethical regulations for animal testing and research. The animal experiments were performed in accordance with the *Guide for the Care and Use of Laboratory Animals* (National Academies Press, 2011). All animals were maintained under specific pathogen-free conditions and used according to protocols approved by the IACUC of the University of Pittsburgh.

## Results

### Demographics

**Fig. 1:**
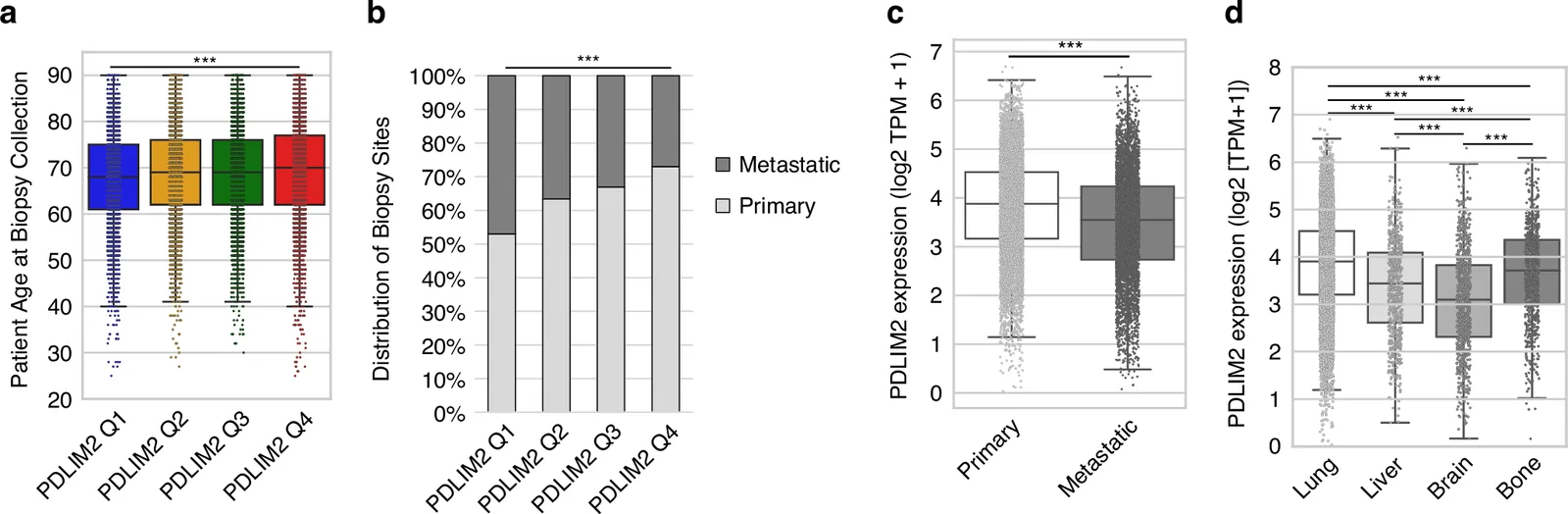
Low PDLIM2 expression is associated with decreased patients’ age and with LUAD metastasis. **a** Column shows that low *PDLIM2* expression was associated with decreased age of LUAD patients. **b** Column shows that low *PDLIM2* expression was detected less frequently in primary LUADs. **c** *PDLIM2* expression was significantly higher in primary LUADs. **d** Significantly lower *PDLIM2* expression was detected in the tumors in metastatic sites compared to the lung.

The demographics and smoking status/history of our 15,765 LUAD patients are listed (Supplemental Table 1). While high *PDLIM2* expression (upper quartile/Q4 in TPM using LUAD tumor expression cutoffs) demonstrated a statistically significant association with increased age of patients (median age Q4: 70 vs Q1: 68; *p* < 0.001), the numerical difference is too small to be clinically relevant (Fig. 1a and Supplemental Table 2). Interestingly, increased *PDLIM2* expression occurred more frequently in primary/local biopsies (Q4: 73.1% vs Q1: 53.0%; *p* < 0.001) and demonstrated a consistent trend across all quartiles (Q4: 73.1%, Q3: 66.9%, Q2: 63.4%, Q1: 53.0%) (Fig. 1b and Supplemental Table 2). Consistently, median *PDLIM2* expression was higher in primary LUADs (Fig. 1c), and LUAD tumors in the lung demonstrated higher transcripts per million (TPM) *PDLIM2* expression compared to bone, liver, and brain sites (16.8 vs 14.7 vs 12.3 vs 10.1, *p* < 0.001, respectively) (Fig. 1d and Supplemental Table 3).

### Molecular alterations

**Fig. 2:**
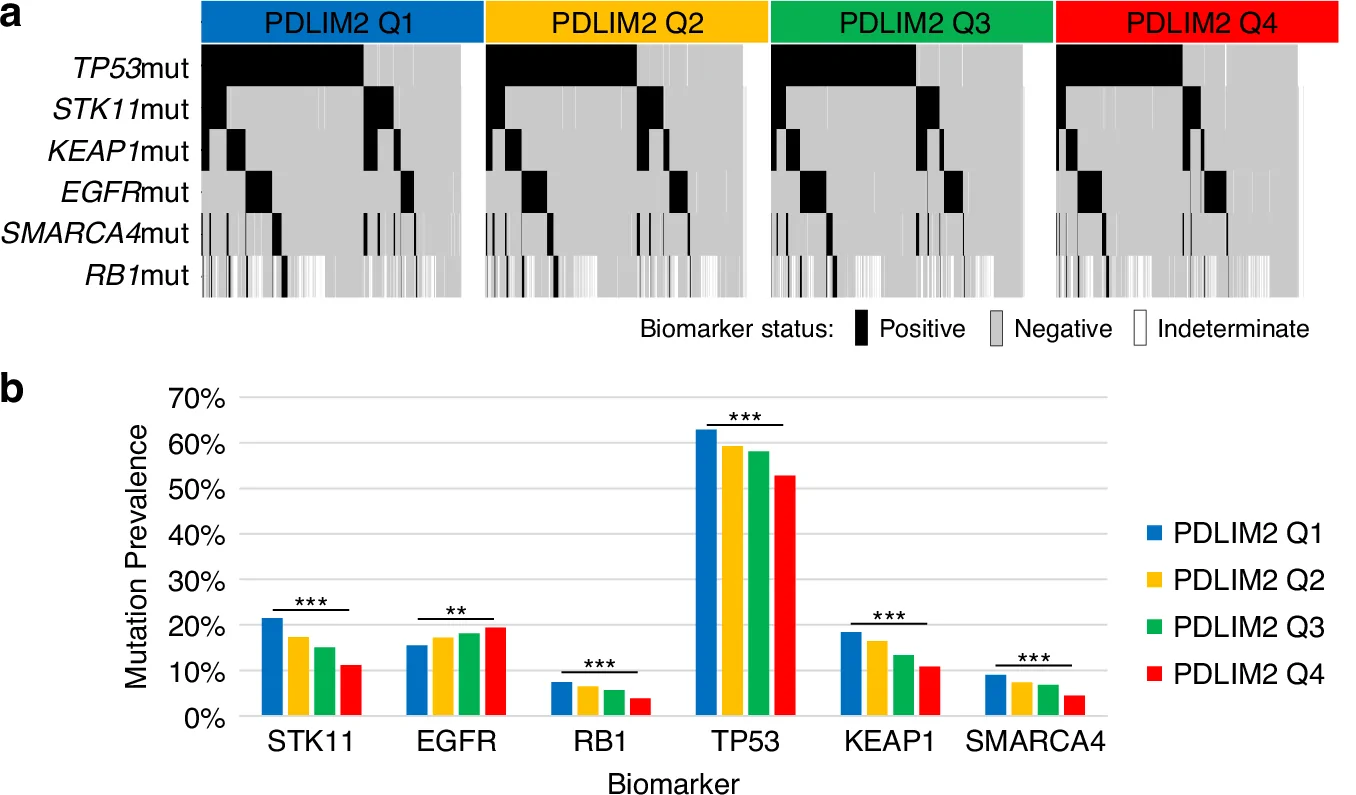
Molecular alteration stratified by PDLIM2 expression quartiles in LUAD. **a** Heatmap shows the correlation of low *PDLIM2* expression with high alteration rates of the tumor suppressor genes. **b** Column shows the correlation of low *PDLIM2* expression with high alteration rates of the tumor suppressor genes.

DNA-seq data showed that high *PDLIM2* expression was correlated with low alteration rates of the tumor suppressor genes *RB1* (Q4: 3.8% vs Q1: 7.5%)*, TP53* (Q4: 52.8% vs Q1: 62.9%*)*, *SMARCA4* (Q4: 4.5% vs Q1: 9.1%), *STK11* (Q4: 11.2% vs Q1: 21.5%,), and *KEAP1* (10.9% vs 18.4%) (all *p* < 0.001); but an opposite pattern was observed for the mutations of the proto-oncogene *EGFR* (Q4: 19.4% vs Q1: 15.5%, *p* < 0.01) (Fig. 2a, b, and Supplemental Table 4). This consistent association between higher *PDLIM2* expression and lower mutation rates in key tumor suppressor genes suggests that preserved *PDLIM2* marks less aggressive genomic profiles in LUAD. No statistical differences were observed for the mutations of the proto-oncogenes *NFE2L2* (Q4: 2.1% vs Q1: 1.9%, *p* = 0.75)*, RAS* (*KRAS*, Q4: 34.2% vs Q1: 37.3%, *p* = 0.34; *HRAS*, Q4: 0.1% vs Q1: 0.4%, *p* = 0.34; *NRAS*, Q4: 0.6% vs Q1: 0.9%, *p* = 0.76) and *BRAF* (Q4: 5.8% vs Q1: 5.2%, *p* = 0.98).

**Fig. 3:**
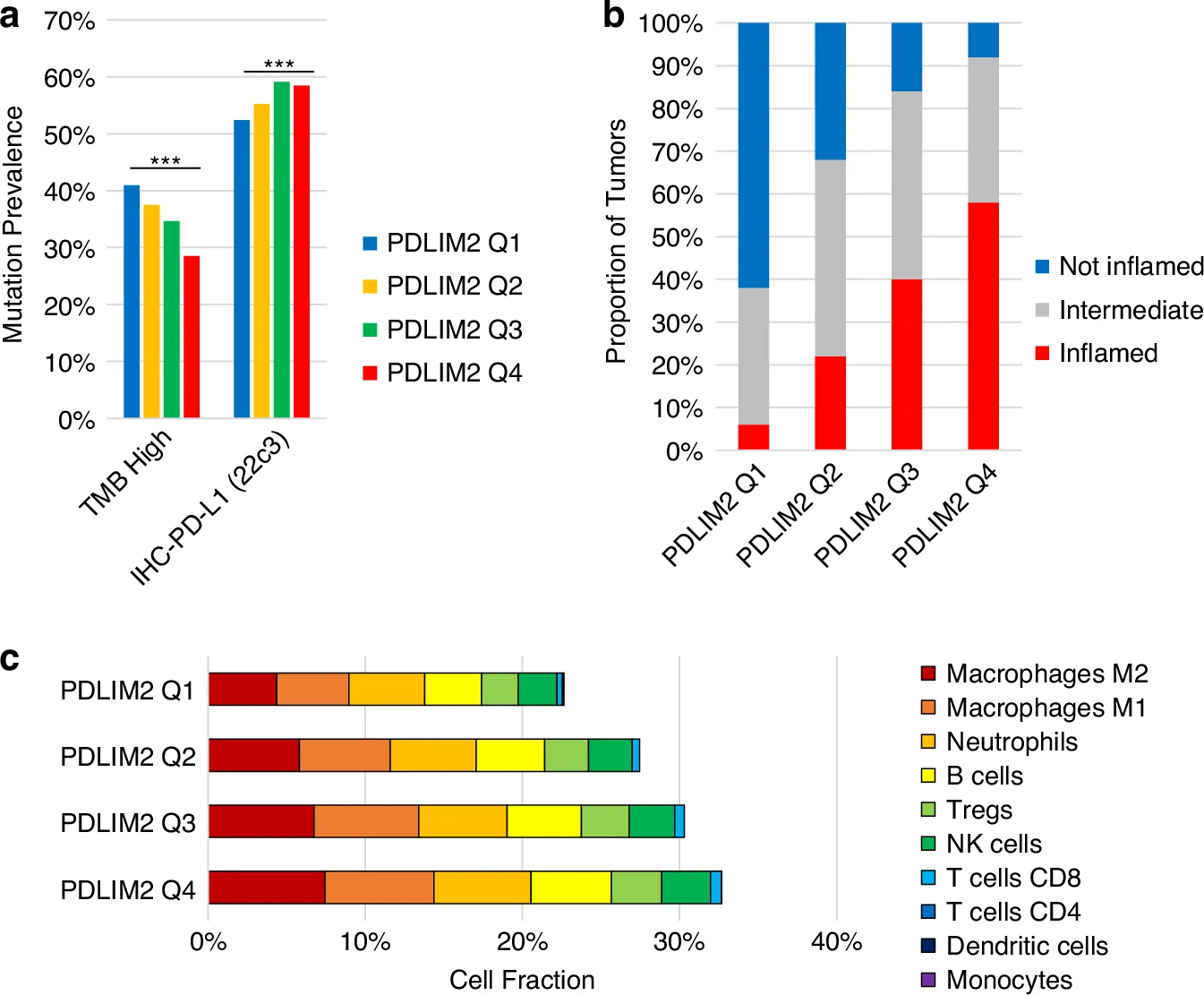
Low *PDLIM2* expression was associated with lower PD-L1 expression on LUAD and decreased immune infiltrate across immune populations. **a** Low *PDLIM2* expression was associated with decreased PD-L1+ but increased TMB-High status. **b** Column shows the correlation of low *PDLIM2* expression with decreased T cell-inflamed scores. **c** Low *PDLIM2* expression was correlated with low tumor infiltration of immune cells.

RNA-seq plus IHC data showed that high *PDLIM2* expression was associated with increased PD-L1+ (Q4: 58.4% vs Q1: 52.4% *p* < 0.001) and decreased TMB-high status (28.5% vs 40.9%, *p* < 0.001) (Fig. 3a and Supplemental Table 5). Furthermore, high *PDLIM2* expression correlated with significantly increased T cell-inflamed scores (Q4: 58% vs Q1: 6% inflamed *p* < 0.001) (Fig. 3b and Supplemental Table 6) and greater tumor infiltration of immune cells including NK cells (Q4: 3.11% vs Q1: 2.48%), CD8 T cells (0.70% vs 0.33%), CD4 T cells (0.01% vs 0.00%), B cells (5.11% vs 3.60%), macrophages (M1: 6.94% vs 4.60%; M2 7.43% vs 4.36%), and neutrophils (6.18% vs 4.83%) (all *p* < 0.008) (Fig. 3c, and Supplemental Fig. 1 and Table 7).

### Patient survival

**Fig. 4:**
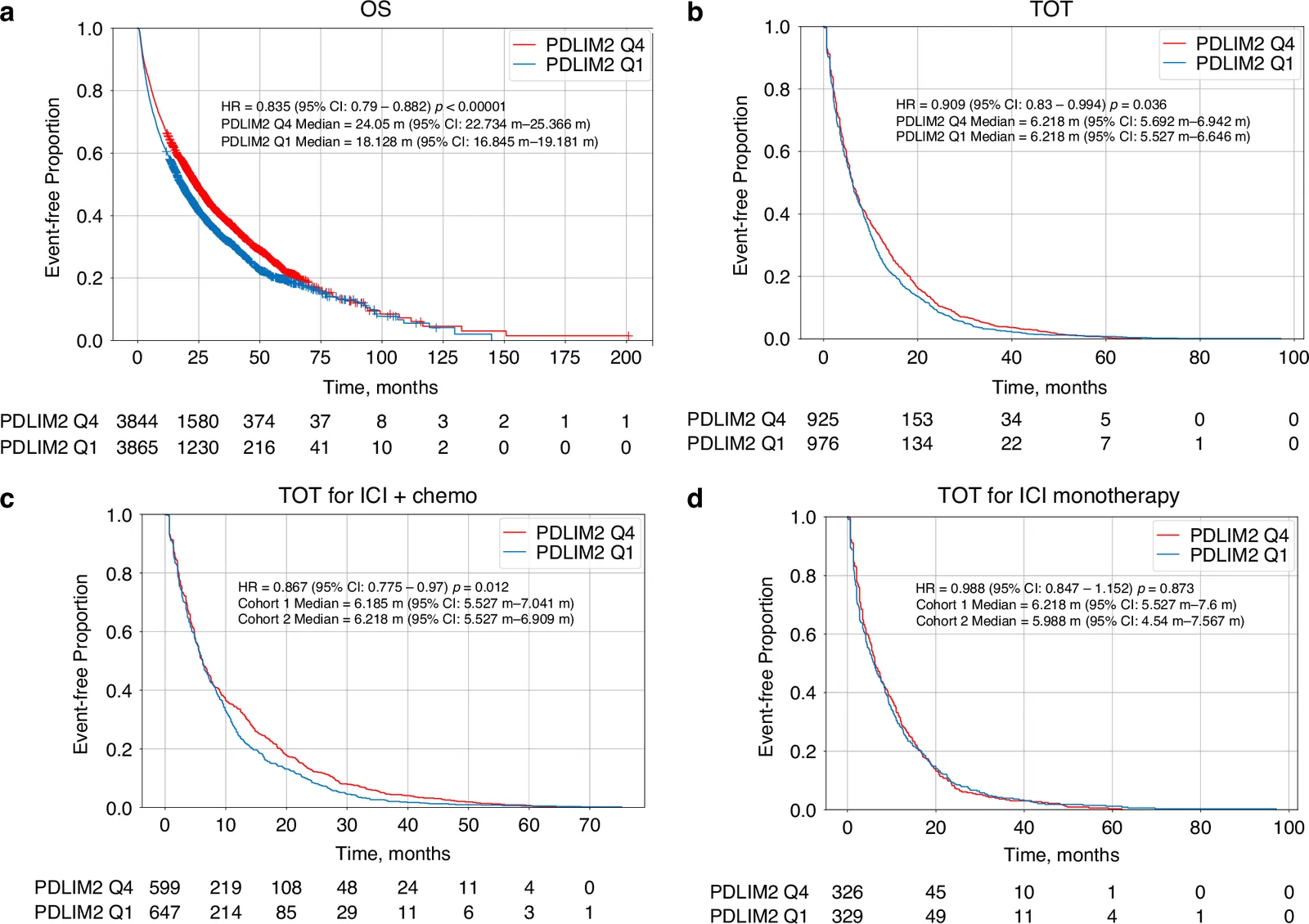
Low *PDLIM2* is associated with poor patient survival. **a** Low *PDLIM2* expression was associated with improved overall survival. **b** Low *PDLIM2* expression was associated with decreased time on pembrolizumab alone or combined with chemotherapy (carboplatin or cisplatin) treatments. **c** Low *PDLIM2* expression was associated with decreased time on the combinational treatment of pembrolizumab and chemotherapy(carboplatin or cisplatin). **d** There was no significant association of *PDLIM2* expression with time on pembrolizumab monotherapy.

Increased *PDLIM2* expression was associated with improved overall survival (OS) (median survival Q4: 24.05 vs Q1: 18.128 months; *p* < 0.00001; Hazard ratio [HR] = 0.835, 95% Confidence Interval [CI] 0.79–0.882) (Fig. 4a). Despite similar median treatment times, higher *PDLIM2* expression was also associated with improved time on treatment (TOT) among patients receiving pembrolizumab alone or with chemotherapy (Q4: 6.218 vs Q1: 6.218 months; *p* = 0.036; HR = 0.909 95% CI 0.830–0.994) (Fig. 4b). The association was statistically significant on the combinational immunochemotherapy (Q4: 6.185 months vs Q1: 6.218 months; *p* = 0.012; HR = 0.867 95% CI 0.775–0.97) with greater separation seen after the median time (Fig. 4c). No statistical difference was detected for patients having pembrolizumab monotherapy (Q4: 6.218 vs Q1: 5.988 months; *p* = 0.873; HR = 0.988 95% CI 0.847–1.152) (Fig. 4d). In a multivariate Cox model adjusting for age, sex, PD-L1 IHC, genetic alterations and treatment group, higher PDLIM2 expression continued to confer improved OS (*p* < 0.0001; HR = 0.88 95% CI 0.83–0.93) (Supplemental Table 8).

### nanoPDLIM2 treatment in mouse syngeneic LUAD models

**Fig. 5:**
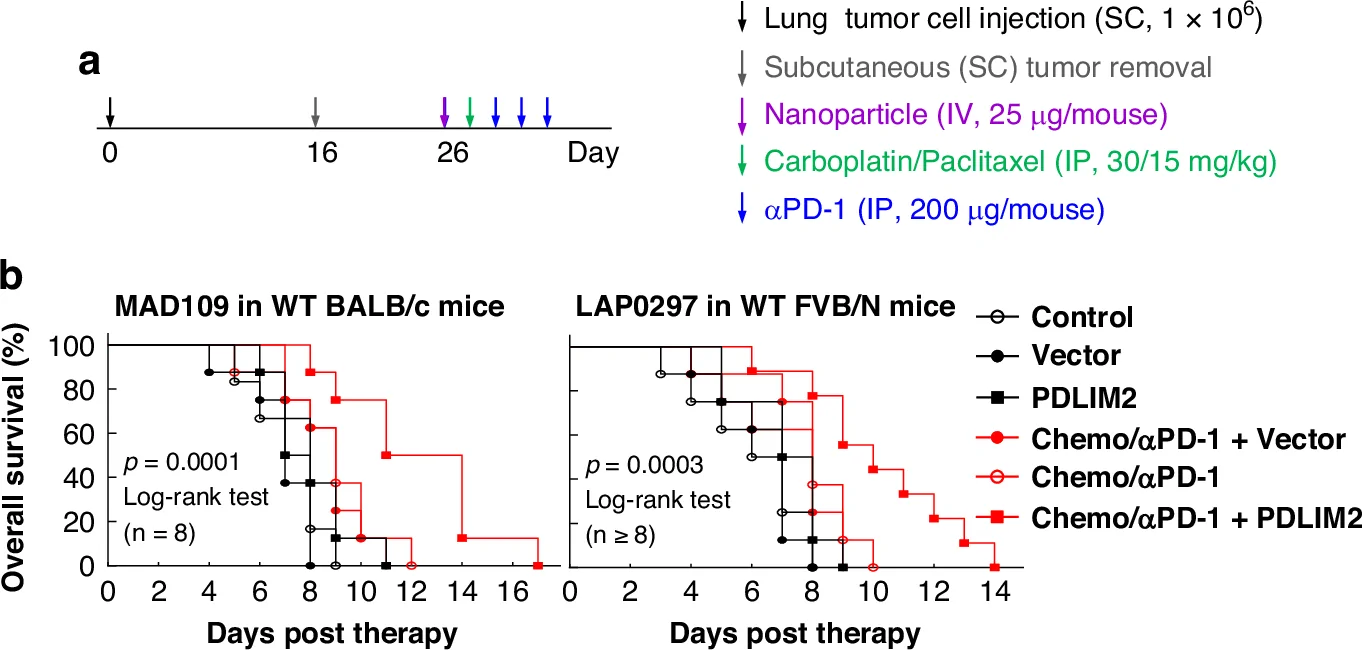
Efficacy and synergy of nanoPDLIM2, PD-1 antibodies and chemo drugs. **a** Schedule of syngeneic mouse model of metastatic lung cancer and treatment. **b** Overall survival of mice with metastatic lung cancer after treatment.

Two syngeneic mouse models (MAD109 and LAP0297) of aggressive metastatic lung tumor, in which all mice die of lung cancers metastasized from s.c injected lung tumors, were used to test whether nanoPDLIM2 enhances the efficacy of combined PD‑1 blockade and chemotherapy. Mice with metastasized lung tumors in each model were randomly divided into six groups for the different treatments listed in Fig. 5. Each group contained 8 mice, except for the triple combination treatment group in LAP0297 lung tumor model, which had 9 mice. Both models showed that even one dose of nanoPDLIM2 tripled mouse OS time improved by anti-PD-1 and chemo-drug combination therapy (*p* = 0.0001 or 0.0003, respectively), as evidenced by the median OS time post treatment: 7 days for non-treatment or nano-vector groups and 7.5 days for nanoPDLIM2 groups in both models, 9 and 8 days for chemoimmunotherapy groups and 12.5 and 10 days for triple-combination therapy groups in the MAD109 and LAP0297 models, respectively. Note, lung cancer cell lines MAD109 and LAP0297 were originally developed from LUAD in a BALB/c mouse and an FVB/N mouse, respectively, and *PDLIM2* is repressed in both mouse lung tumor cell lines used in the models [5, 7, 32, 33].

## Discussion

Our study highlights the importance of *PDLIM2* expression as a biomarker in LUAD, where higher expression is associated with longer survival and improved outcomes in patients receiving chemoimmunotherapy. LUAD with elevated *PDLIM2* levels demonstrated fewer alterations linked to poor outcomes, such as *TP53*, *STK11* and *KEAP1*, alongside enhanced immune cell infiltration and greater PD-L1 positivity, suggesting a more immunologically active tumor microenvironment. These findings not only deepen our understanding of the molecular and immune characteristics driving outcomes in LUAD but also hold significant therapeutic implications. nanoPDLIM2, an emerging therapy with promising preclinical results in both primary and metastatic LUADs, could potentially leverage these insights to target LUAD more effectively. By establishing PDLIM2 as an independent prognostic factor in a multivariable model and identifying its association with improved outcomes specifically in patients receiving chemoimmunotherapy, this study underscores its relevance in advancing personalized treatment strategies for NSCLC and supporting the development of novel therapeutic modalities.

The higher *PDLIM2* expression observed in lung tumors compared to common metastatic sites such as the liver and bone may reflect that further *PDLIM2* repression contributes to lung tumor metastasis, particularly metastasizing out of the lung. It may also reflect the different tumor microenvironment. In this regard, *PDLIM2* is known to be expressed highest in the lung under normal conditions, and *PDLIM2* in primary lung tumors is dramatically decreased in comparison to normal lung cells [5, 6, 37–39]. DNA methylation may play a key role in modulating *PDLIM2* expression across different tumor sites. Preclinical models in colorectal and breast cancer have demonstrated that DNA hypermethylation can suppress *PDLIM2* expression [12, 13]. Similarly, an analysis of TCGA data in NSCLC revealed an inverse correlation between the DNA methylation and histone deacetylation of the *PDLIM2* promoter and its mRNA expression [5]. The different hypoxia within tumors at different sites may also be involved, as the *PDLIM2* expression could be repressed by oxidative stress-activated transcription repressor BACH1 [40].

The lower prevalence of mutations in the tumor suppressor genes *STK11*, *TP53*, *KEAP1*, *RB1*, and *SMARCA4* in *PDLIM2*-high tumors may highlight a favorable tumor profile, as these genes are often associated with poor prognosis and treatment resistance in NSCLC [41–46]. *STK11* and *KEAP1* and *TP53* mutations are resistant to immunotherapy and may mediate their effects on immunotherapy resistance by repressing *PDLIM2*. Exploratory analysis of KEYNOTE-189 demonstrated that the addition of pembrolizumab to chemotherapy did not improve OS or progression-free survival (PFS) in *KEAP1* or *STK11* mutant subgroup, while the wildtype cohorts showed improved OS and PFS with chemotherapy/immunotherapy combination in NSCLC [47]. Similarly, mutations in *RB1* and *SMARCA4* are associated with rare, aggressive histology and poor prognosis of tumors, such as *SMARCA4*-deficient thoracic sarcomas and small cell transformation [48, 49]. Thus, their lower prevalence in *PDLIM2*-high tumors suggests that high *PDLIM2* expression may correspond to a less aggressive molecular profile.

We noted significant associations between *PDLIM2* expression and immunological factors in LUAD. High *PDLIM2* expression correlated with increased PD-L1 positivity, suggesting that *PDLIM2* may be a marker of an immune-receptive tumor microenvironment. *PDLIM2* expression may complement existing biomarkers in risk stratification frameworks to help identify patients who may derive greater benefit from chemoimmunotherapy. Exploratory analysis of the POSEIDON trial highlighted that dual immunotherapy of durvalumab (anti-PD-L1) and tremelimumab (anti-cytotoxic T lymphocyte-associated antigen 4, CTLA-4) in combination with chemotherapy led to an OS benefit in Immuno-oncology (IO)-resistant subgroups such as *STK11* mutant NSCLC [50]. Conversely, high *PDLIM2* expression was associated with a decreased TMB-high status, which portends decreased immunotherapy response due to the lower neoantigen load stimulating immune recognition. In LUAD, increased *PDLIM2* expression correlated with higher T cell-inflamed scores, along with enhanced infiltration of immune cells, including NK cells, T cells, macrophages, neutrophils, and B cells. This indicates that *PDLIM2* is associated with a more immunologically active tumor microenvironment, though whether this reflects an independent mechanistic role or co-enrichment with other immune-favorable features remains to be determined. In this regard, *PDLIM2* is known to be involved in antigen presentation and T-cell activation, potentially contributing to enhanced anti-tumor immune responses with increased expression [5, 6]. In further support of this, preclinical ICI treated NSCLC models noted increased tumor infiltrating lymphocytes and improved outcomes with the addition of *PDLIM2* therapy, highlighting the synergistic effect [5, 6] (Fig. 5). Indeed, our syngeneic LUAD models demonstrate enhanced efficacy of chemoimmunotherapy. We did not isolate a PDLIM2-dependent effect on PD-1 blockade alone in this highly aggressive model, as it allows only a single treatment cycle.

We demonstrated that higher PDLIM2 expression was associated with improved OS in LUAD patients and with improved TOT among patients receiving pembrolizumab combined with platinum-based chemotherapy. No significant benefit was observed in patients receiving pembrolizumab monotherapy, suggesting that the clinical relevance of PDLIM2 is most pronounced in the chemoimmunotherapy setting and does not support an independent predictive role for PD-1 blockade alone. Notably, the prognostic significance of PDLIM2 was confirmed in our multivariable OS model, consistent with a prior multi-database assessment of NSCLC including TCGA. This prognostic effect may be attributed to its tumor suppressor activity through repression of the NF-κB and STAT3 signaling pathways, which promote cancer progression and therapy resistance [5, 6, 11–13].

Our study has limitations, including its retrospective design, the heterogeneity of the Caris study population, and the lack of detailed information on treatment sequences within the Caris dataset. Additionally, we did not have PDLIM2 immunohistochemistry (IHC) data to validate our gene expression findings or confirm the functional activity of *PDLIM2*. Furthermore, the gene expression thresholds were derived internally from this dataset, which may impact generalizability. Our preclinical nanoPDLIM2 studies were also performed exclusively in chemoimmunotherapy models, which limits our ability to definitively attribute the observed benefit to immunotherapy alone. Despite these limitations, our study emphasizes biomarker discovery, underscoring the need for future prospective studies to validate the independent prognostic value of PDLIM2 expression and to clarify any potential predictive role, particularly in the context of established immunosuppressive alterations such as *STK11* and *KEAP1* mutations and PD-L1 co-expression, in NSCLC.

In summary, our study demonstrates that in NSCLC-adenocarcinoma, high *PDLIM2* expression correlates with a favorable molecular profile, including decreased mutations in tumor suppressor genes such as *STK11*, *TP53*, *KEAP1*, *RB1*, and *SMARCA4*. Additionally, high *PDLIM2* levels are associated with increased PD-L1, enhanced immune cell infiltration and higher T cell-inflamed scores, indicating a more active antitumor immune microenvironment. The molecular and cellular landscapes may contribute to the improved overall survival observed in patients with high *PDLIM2* expression. These findings highlight the potential of *PDLIM2* as a prognostic biomarker and a candidate target for enhancing chemoimmunotherapy efficacy, underscoring the need for prospective validation to fully define its independent prognostic and predictive role in NSCLC.

## Supplementary information

Supplementary information

## Data Availability

The datasets generated and/or analyzed during the current study are available from the corresponding author upon reasonable request. The deidentified sequencing data were obtained from Caris Life Sciences. Qualified researchers can apply for access to these summarized data by contacting Andrew Elliott, PhD, and signing a data-usage agreement. Animal study raw data are available upon reasonable request from the corresponding author.
