# Supplementary material for: Exploring PDLIM2 as a prognostic biomarker and therapeutic target in lung adenocarcinoma
Source: Br J Cancer. 2026 Aug 8;135(10):1686–93. doi: 10.1038/s41416-026-03570-3 (PMC13740284; doi:10.1038/s41416-026-03570-3)
Supplement: Supplementary file 1 [file 41416_2026_3570_MOESM1_ESM.docx]

**Supplemental Figures/Tables**

**Supplemental Figure 1. Increasing immune infiltrate was associated with increased *PDLIM2* expression across immune populations**

**Supplemental Table 1. Patient demographics by PDLIM2 quartile**

| **PDLIM2 Subgroup** | | **Q1** | **Q2** | **Q3** | **Q4** | **Statistic** | ***p*-value** |
| --- | --- | --- | --- | --- | --- | --- | --- |
| **Sex** | **Male** | **47.4%** | **44.9%** | **45.7%** | **44.4%** | **chi-square** | **0.039** |
|  | **Female** | **52.6%** | **55.1%** | **54.3%** | **55.6%** |  |  |
| **Race** | **White** | **63.8%** | **65.3%** | **66.9%** | **65.1%** | **chi-square** | **0.00004** |
|  | **Black or African American** | **13.0%** | **11.1%** | **11.5%** | **10.5%** |  |  |
|  | **Asian or Pacific Islander** | **2.6%** | **3.9%** | **3.4%** | **4.2%** |  |  |
|  | **Other** | **2.6%** | **2.7%** | **2.8%** | **3.2%** |  |  |
|  | **Unknown** | **18.0%** | **17.0%** | **15.5%** | **17.1%** |  |  |
| **Ethnicity** | **Hispanic or Latino** | **6.1%** | **6.6%** | **5.7%** | **6.6%** | **chi-square** | **0.034** |
|  | **Not Hispanic or Latino** | **76.1%** | **76.0%** | **78.6%** | **77.3%** |  |  |
|  | **Unknown** | **17.9%** | **17.4%** | **15.7%** | **16.1%** |  |  |
| **Smoking Status** | **Current Smoker** | **13.7%** | **13.4%** | **13.1%** | **14.6%** | **chi-square** | **0.0006** |
|  | **Light Smoker (<15 packs_yr)** | **6.8%** | **6.1%** | **7.0%** | **5.4%** |  |  |
|  | **Lifelong Non-smoker** | **0.7%** | **0.6%** | **0.6%** | **1.2%** |  |  |
|  | **Unknown** | **78.9%** | **80.0%** | **79.3%** | **78.8%** |  |  |

**Supplemental Table 2. Low PDLIM2 expression is associated with decreased patient age and occurs less frequently in primary/local LUAD in the lung**

|  | **Q1** | **Q2** | **Q3** | **Q4** | **Statistic** | ***p*-value** |
| --- | --- | --- | --- | --- | --- | --- |
| **Count (N)** | **3942** | **3941** | **3941** | **3941** |  |  |
| **Median Age  (Range)** | **68  (25 – 90+)** | **69 (27 – 90+)** | **69  (30 – 90+)** | **70  (25 – 90+)** | **Kruskal-Wallis** | **< 0.001** |
| **Primary/local biopsy** | **53.0%** | **63.4%** | **66.9%** | **73.1%** | **chi-square** | **< 0.001** |

**Supplemental Table 3. Low PDLIM2 expression is associated with LUAD out of the lung**

|  | **Lung** | **Liver** | **Brain** | **Bone** | **Statistic** | ***p*-value** |
| --- | --- | --- | --- | --- | --- | --- |
| **Average PDLIM2 (TPM)** | **16.8** | **12.3** | **10.1** | **14.7** | **Mann-Whitney U** | **< 0.001** |

**Supplemental Table 4. Genetic mutation rates in LUAD with different PDLIM2 expression levels**

|  | **Q1** | **Q2** | **Q3** | **Q4** |
| --- | --- | --- | --- | --- |
| **STK11** | **21.5%** | **17.3%** | **15.1%** | **11.2%** |
| **EGFR** | **15.5%** | **17.2%** | **18.2%** | **19.4%** |
| **RB1** | **7.5%** | **6.5%** | **5.7%** | **3.8%** |
| **TP53** | **62.9%** | **59.3%** | **58.1%** | **52.8%** |
| **KEAP1** | **18.4%** | **16.4%** | **13.4%** | **10.9%** |
| **SMARCA4** | **9.1%** | **7.4%** | **6.8%** | **4.5%** |

**Supplemental Table 5. Low PDLIM2 expression is associated with high TMB and low PD-L1**

|  | **Q1** | **Q2** | **Q3** | **Q4** |
| --- | --- | --- | --- | --- |
| **TMB High** | **40.9%** | **37.5%** | **34.7%** | **28.5%** |
| **IHC-PD-L1** | **52.4%** | **55.2%** | **59.1%** | **58.4%** |

**Supplemental Table 6. Low PDLIM2 expression is associated with T-cell inflamed score of LUAD**

| **T-cell inflamed** | **Not inflamed** | **Intermediate** | **Inflamed** |
| --- | --- | --- | --- |
| **PDLIM2 Q1** | **62%** | **32%** | **6%** |
| **PDLIM2 Q2** | **32%** | **46%** | **22%** |
| **PDLIM2 Q3** | **16%** | **44%** | **40%** |
| **PDLIM2 Q4** | **8%** | **34%** | **58%** |

**Supplemental Table 7. Low PDLIM2 expression is associated with immune cell infiltration in LUAD**

|  | **Q1** | **Q2** | **Q3** | **Q4** | ***p*-value** |
| --- | --- | --- | --- | --- | --- |
| **Tregs** | 2.33% | 2.81% | 3.04% | 3.20% | <0.001 |
| **Dendritic cells** | 0.11% | 0.00% | 0.00% | 0.00% | <0.001 |
| **Monocytes** | 0.00% | 0.00% | 0.00% | 0.00% | <0.001 |
| **NK cells** | 2.48% | 2.77% | 2.88% | 3.11% | <0.002 |
| **T cells CD8** | 0.33% | 0.47% | 0.63% | 0.70% | <0.003 |
| **T cells CD4** | 0.00% | 0.00% | 0.00% | 0.01% | <0.004 |
| **B cells** | 3.60% | 4.35% | 4.73% | 5.11% | <0.005 |
| **Macrophages M2** | 4.36% | 5.80% | 6.73% | 7.43% | <0.006 |
| **Macrophages M1** | 4.60% | 5.79% | 6.66% | 6.94% | <0.007 |
| **Neutrophils** | 4.83% | 5.47% | 5.64% | 6.18% | <0.008 |

**Supplemental Table 8. Multivariate cox regression model demonstrating high PDLIM2 expression is associated with improved overall survival in LUAD when controlling for age, sex, genetic markers and treatment groups.**

| Feature | HR | Lower 95% | Upper 95% | P-value |
| --- | --- | --- | --- | --- |
| KEAP1 | 1.40 | 1.29 | 1.53 | <0.0001 |
| TP53 | 1.29 | 1.21 | 1.38 | <0.0001 |
| STK11 | 1.25 | 1.15 | 1.36 | <0.0001 |
| Sex (Male) | 1.16 | 1.09 | 1.24 | <0.0001 |
| ROS1 | 1.13 | 0.79 | 1.63 | 0.5037 |
| Age (>= 65 years) | 1.11 | 1.04 | 1.19 | 0.0016 |
| KRAS | 1.05 | 0.98 | 1.13 | 0.1706 |
| PDL1 (22c3) | 1.00 | 0.94 | 1.07 | 0.8838 |
| BRAF | 0.97 | 0.84 | 1.11 | 0.6292 |
| EGFR | 0.96 | 0.85 | 1.07 | 0.422 |
| **PDLIM2 Q4** | 0.88 | 0.83 | 0.93 | <0.0001 |
| Chemo + ICI | 0.85 | 0.78 | 0.92 | <0.0001 |
| RAS/RAF targeted | 0.84 | 0.69 | 1.03 | 0.0888 |
| Non-RAS/RAF targeted | 0.79 | 0.70 | 0.90 | 0.0002 |
| ICI (mono) | 0.69 | 0.61 | 0.78 | <0.0001 |
| ALK | 0.65 | 0.50 | 0.83 | 0.0006 |
| Chemo (mono) | 0.63 | 0.58 | 0.68 | <0.0001 |
